# A practical guidance on the prevention and treatment of childhood respiratory syncytial virus infection in Kurdistan

**DOI:** 10.3389/fped.2025.1551734

**Published:** 2025-03-13

**Authors:** Azad A. Haleem, Azhar Alsaqee, Lana A. Dizayi, Sasan L. Hanna, Abbas A. Rabaty, Serdar Pedawi, Aso F. Salih

**Affiliations:** ^1^ Department of Pediatrics, College of Medicine, University of Duhok, Duhok, Kurdistan, Iraq; ^2^ Neonatology and Intensive Care Unit, College of Medicine, Hawler Medical University, Erbil, Kurdistan, Iraq; ^3^ College of Medicine, Salaheddin University, Erbil, Kurdistan, Iraq; ^4^ Department of Pediatrics, College of Medicine, Hawler Medical University, Erbil, Kurdistan, Iraq; ^5^ Division of Pediatric Cardiology, Heevi Pediatric Teaching Hospital, Azadi Heart Center, Duhok, Kurdistan, Iraq; ^6^ Clinical Science Department, Children’s Heart Hospital, College of Medicine, Sulaimani University, Sulaymaniyah, Kurdistan, Iraq; ^7^ Department of Medical Laboratory Technology, Faculty of Health Sciences, Qaiwan International University, Sulaymaniyah, Kurdistan, Iraq

**Keywords:** respiratory syncytial virus, palivizumab, immunoprophylaxis, pediatric respiratory care, Kurdistan

## Abstract

Without an available vaccine in Kurdistan, Respiratory Syncytial Virus (RSV) infection threatens younger children, burdens the healthcare system and contributes to household expenditure on health. Immunoprophylaxis remains the only preventive option in Kurdistan. Expert pediatricians recommend palivizumab to children at RSV risk; particularly infants (1) born within 29 gestational weeks and <1-year-old at RSV season start, (2) born premature (>29 gestational weeks) at risk for RSV hospitalization, (3) with congenital lung disease requiring oxygen therapy for >1 month and are <2 years old at RSV season start, and (4) with hemodynamically significant congenital heart disease and acyanotic heart and who are <2 years of age at RSV season start. We call onto health authorities to support palivizumab immunoprophylaxis to all children at risk for RSV.

## 1 Introduction

Respiratory Syncytial Virus (RSV) infection in children can cause respiratory distress and long-term complications, if it spreads to the lower respiratory tract (1). In fact, RSV is responsible for more than 80% of lower respiratory tract infections in infants below 1 year of age and is the leading cause of pediatric bronchiolitis (1).

Risk for RSV infection and transmission is exacerbated by young age (<6 weeks), male sex, day care attendance, exposure to passive smoking in the home, and crowded living conditions (2). Preterm infants, particularly those born before 32 weeks of gestation, are at particularly higher risk of severe RSV disease due to under-developed immune system and to very small airways (3, 4). RSV-related mortality rate is also higher among infants with trisomy 21, even in the absence of other RSV risk factors (5). Chronic lung disease (CLD) and congenital heart disease (CHD) also increase the susceptibility to severe RSV disease and complications, such as hepatitis (6, 7).

The seriousness of RSV is accentuated by the unavailability of safe and effective vaccination (1, 8), and passive prophylaxis remains the only option for prevention of RSV in high-risk infants in Kurdistan and the region (1). Palivizumab was approved by the United States (US) Food and Drug Administration (FDA) in 1998 as a monoclonal anti-RSV antibody targeting the virus F protein, a highly conserved structure, making it effective against all RSV subtypes (3). Palivizumab protects high-risk infants against the progression of RSV infection to bronchiolitis and viral pneumonia (9, 10).

Updated international guidelines, based on data from epidemiological studies and from clinical experience, recommend the use of palivizumab for immunoprophylaxis in subsets of children at high risk for developing severe RSV (11). These 2014 guidelines define prematurity at the cut-off gestational age of 29 weeks 0 day and reluctantly recommend palivizumab immunoprophylaxis in the second year of life, except in distinct cases and based on pediatrician's clinical opinion. While in their broad lines these guidelines might serve global populations, fine-tuning of the general recommendations is warranted to fit with particular demographic, socio-economic, environmental and healthcare structures (11). Recently in 2023, another monoclonal antibody, nirsevimab, was FDA-approved for use in all infants and children at their first RSV season; administered as a single shot of 50 or 100 mg depending on weight, with around 5-month efficacy (12, 13).

In the Middle East and North Africa (MENA) region, RSV infection prevalence is estimated at 24.4%, in line with the global prevalence of 22% (14). Across the MENA region, the most severe form, RSV type A is threefold as widespread as the less lethal RSV type B virus (15). Among children with pneumonia in Iraq in 2014–2015, RSV type B was predominant (14% of pneumonia cases), compared to RSV type A (8% of cases) (16). Data on RSV epidemiology and clinical management in Kurdistan are scarce. A 2018 study in Erbil city, capital of the Kurdistan region of Iraq, showed that 20% of viral respiratory tract infections in children were due to RSV and that around 16% of children present with co-infections (17). The prevention of RSV-related illnesses and hospital admissions is hindered by the relatively elevated cost of palivizumab (18), estimated at the equivalent of about US $200 per dose in Kurdistan. Medical priorities, dispatching and distribution of palivizumab (the only RSV prophylactic agent available in the region), and the purchasing potential of families make access to immunoprophylaxis erratic and limited. This manuscript, published by leading pediatricians in Kurdistan, provides clear clinical practice guidelines for the management of RSV in Kurdistan and aims to serve as a manual for pediatricians in the region. Additionally, it summarizes the clinical opinion of experts in their field to be presented to health authorities in Kurdistan and centrally in Iraq for the endorsement of recommendations for RSV prevention and management.

## 2 Depicting the situation in Kurdistan

### 2.1 Methodology

A panel of six pediatricians with extensive experience in RSV management from Kurdistan convened and structured roundtable discussions. In-depth insight was collected from participants, with detailed depiction of the challenging situation in Kurdistan and the struggles of the healthcare system.

Deliberations took place, tackling the start of the RSV season in Kurdistan, the cut-off age for prematurity considered relevant for RSV prophylaxis, as well as the particular cases of infants born with CLD of prematurity, including bronchopulmonary dysplasia (BPD), with CHD, with trisomy 21 and other scenarios.

Clinical practice guidance provided by expert physicians in Kurdistan in this manuscript considers international recommendations, but also variations and regional circumstances special to Kurdistan. The authors encourage staying updated on new literature and recommendations for the management of RSV.

### 2.2 RSV season

According to the World Health Organization, “a disease outbreak is the occurrence of cases of disease in excess of what would normally be expected in a defined community, geographical area or season” (19). In Kurdistan, seasonal RSV outbreaks usually start around October and end in March. The 5-month season peaks in December and January, coinciding with the cold season (20).

### 2.3 Diagnosis of RSV in Kurdistan

In general, the diagnosis of RSV is clinical, depending on the season of year. Most cases usually have mild bronchiolitis; such mild cases might progress to more severe clinical presentation. For infants/children admitted with moderate-to-severe clinical symptoms (severe tachypnea, fever, respiratory symptoms), the diagnosis of RSV is usually performed by polymerase chain reaction (PCR) to confirm the virus and initiate the appropriate treatment. A PCR diagnosis for a panel of 20 different respiratory viruses costs between $30–40. Chest radiography is sometimes used to rule out other causes of lower respiratory tract infections, such as bacterial pneumonia or cardio-pulmonary diseases (21). Additionally, in severe cases, levels of inflammatory markers (C-reactive protein and D-dimers) are evaluated (22, 23) and an echocardiography is performed to monitor the extra-pulmonary manifestations of RSV on myocardial performance (24, 25). Experts suggest using the Modified Bronchiolitis Cincinnati Score, as applied in a 2021 study (26) to assess RSV disease severity.

### 2.4 Transmission of RSV

RSV infection occurs mostly through community and household exposures, upon close contact with aerosols emitted from an infected individual or with surfaces contaminated with viral particles (27). Although the mode of transmission of RSV has long been established (28, 29), human behavior and practices remain at the forefront of RSV outbreaks (27). Moreover, nosocomial RSV infections also substantially contribute to RSV incidence, especially in lower income countries (30, 31). As recently reviewed, vertical transmission of RSV during pregnancy is possible and has been associated with impaired immunity and hyperactive airways in the newborn (32). Immunoprophylaxis not only attenuates RSV symptoms and complications in infants; it also serves the purpose to limit RSV transmission among infants and to older adults (33). Experts in Kurdistan raised concerns about unsatisfactory hygiene measures in hospitals due to crowdedness and absence of separate infectious or respiratory wards. They request from health authorities support to create separate units for infectious respiratory diseases in hospitals and raising awareness about hand hygiene practices among hospital staff.

### 2.5 Management of RSV in Kurdistan

Currently in Kurdistan, palivizumab is the only available prophylactic and therapeutic option for RSV infection in high-risk children (34); and experts advocate for the use of palivizumab in all infants and children at risk for RSV infection. For patients with RSV infection, supportive care is offered to treat associated symptoms (35). Experts in Kurdistan recommend oxygen therapy if oxygen saturation (SpO_2_) drops below 92%, although the American Academy of Pediatrics (AAP) set a less conservative cutoff of 90% (36). High flow nasal cannula is also an option before mechanical ventilation, which might be required for 5%–20% of patients, depending on their overall health status (35). The use of bronchodilators is controversial, as reviewed in 2009 (35) and, following the AAP recommendation, corticosteroids should be cautiously in patients with bronchospasms or a family history of asthma (36) and not in very young infants (<1 year of age) (37). In Kurdistan, objective positive response among infants older than 6 months motivates maintenance of bronchodilator treatment (mostly salbutamol), but their use in infants below 6 months of age is restrictive. They usually do not use adrenalin (epinephrine) nebulizer, in line with the literature where a clear benefit failed to be demonstrated (35). The use of saline solution (nebulizer) has been endorsed by experts in Kurdistan, as this practice proved beneficial to children with RSV. Pediatricians in Kurdistan are reluctant to use corticosteroids (intravenous route or other modes of administration), in line with global practice (35, 38). However, they do keep a steroid burst as an option for complicated cases and hyperactive airways, as steroid use may be associated with shorter hospital stays (39).

## 3 Guidelines on palivizumab use for RSV hospitalization prevention

Ten years ago, the AAP updated their guidance on palivizumab prophylaxis among infants and children at increased risk of hospitalization due to RSV infection (11). In summary, their recommendations came as follows.

### 3.1 RSV prophylaxis on the basis of prematurity

Palivizumab may be given to infants born before 29 weeks of gestation without CLD of prematurity or CHD and who are below 1 year of age at the RSV season. Infants born premature but beyond week 29 of gestation may be given palivizumab prophylaxis if they present with other conditions that might contribute to increased risk of RSV hospitalization.

In Kurdistan, experts recommend palivizumab prophylaxis for infants born before 34 weeks of gestation and who are younger than 6 months at the start of the RSV season. This more conservative definition of prematurity fits well with RSV risk factors in Kurdistan, such as household crowdedness and multiple young siblings, lower socio-economic status, air pollution, and others. In Kurdistan, 35% of the population is below 15 years of age and 28% are between 15 and 29 years old and close to 14% of the population are illiterate (40, 41). The average household size in Kurdistan was 5.1 per household in 2017 (40), compared to much lower figures in the United States at 2.6 people per household in 2022 (42). Additionally, in 2019, 10% of the Kurdistan workforce was unemployed and figures were higher in Duhok (16%) compared to Erbil or Sulaymaniyah (9%) (43).

### 3.2 RSV prophylaxis on the basis of CLD including BPD

AAP guidelines recommend the use of palivizumab during the first year of life in infants with CLD of prematurity defined as gestational age below 32 weeks who require therapeutic oxygen for at least the first 28 days after birth (11). In Kurdistan, experts recommend palivizumab to infants with CLD (including BPD) who have required oxygen therapy for longer than 1 month and who are below 2 years of age at the start of the RSV season.

### 3.3 RSV prophylaxis on the basis of hemodynamically significant CHD

According to the 2014 AAP guidelines, palivizumab prophylaxis can be administered to children with hemodynamically significant CHD and acyanotic heart and who are below 2 years of age at the onset of RSV season (11). Expert pediatricians in Kurdistan endorse the use palivizumab for all children with hemodynamically significant CHD (regardless of cyanosis status), who are below 2 years of age the start of RSV season.

The less stringent criteria for palivizumab use in Kurdistan stems from the real-life experience of clinicians in Kurdistan as well as from the household and healthcare system structures. As aforementioned, one dose of palivizumab in Kurdistan amounts to US $200 (not covered by the Department of Health, as it is listed as a Category B drug in Iraq); but experts estimate that the cost of illness and the burden on the healthcare system would largely exceed the cost of palivizumab prophylaxis.

## 4 RSV burden

Multiple studies have underscored the multi-level burden of RSV on the patients' immediate and long-term health outcomes, on the family's expenditure on healthcare and on the healthcare system (44, 45). Individuals who were not infected by RSV as infants and children are at a lower risk for hyperactive airways and the development of asthma by the age of 5 years (45, 46). Additionally, RSV infection has been associated with systemic dissemination and with long-term extra-pulmonary manifestations such as cardiac arrhythmias, seizures and hepatitis (in as many as 46%–49% of infants requiring ventilation therapy) (47). While Kurdistan-specific data are virtually inexistent, experts in Kurdistan are concerned with the eventuality of long-term complications in infants and children below 2 years of age who contract RSV and present with severe forms of the disease. Additionally, they estimate an average of US $200 per day in hospital stay out-of-pocket expenditure, reaching US $700 if the patient requires intensive care. This heavily burdens a family's financial status, especially in Kurdistan (41, 43), and given the impact of lower-income countries on the rate of hospital admission for RSV (48). The risk of hospital re-admission for RSV is also non-negligible, with 6.5% of children re-admitted to the hospital for RSV as reported in a recent study (49), increasing the risk of nosocomial infections that may substantially contribute to the burden of RSV-related expenditures and mortality (31, 50, 51) especially that a prior infection may not attenuate the risk for a re-infection (52, 53).

## 5 Actionable recommendations

**Figure 1:**
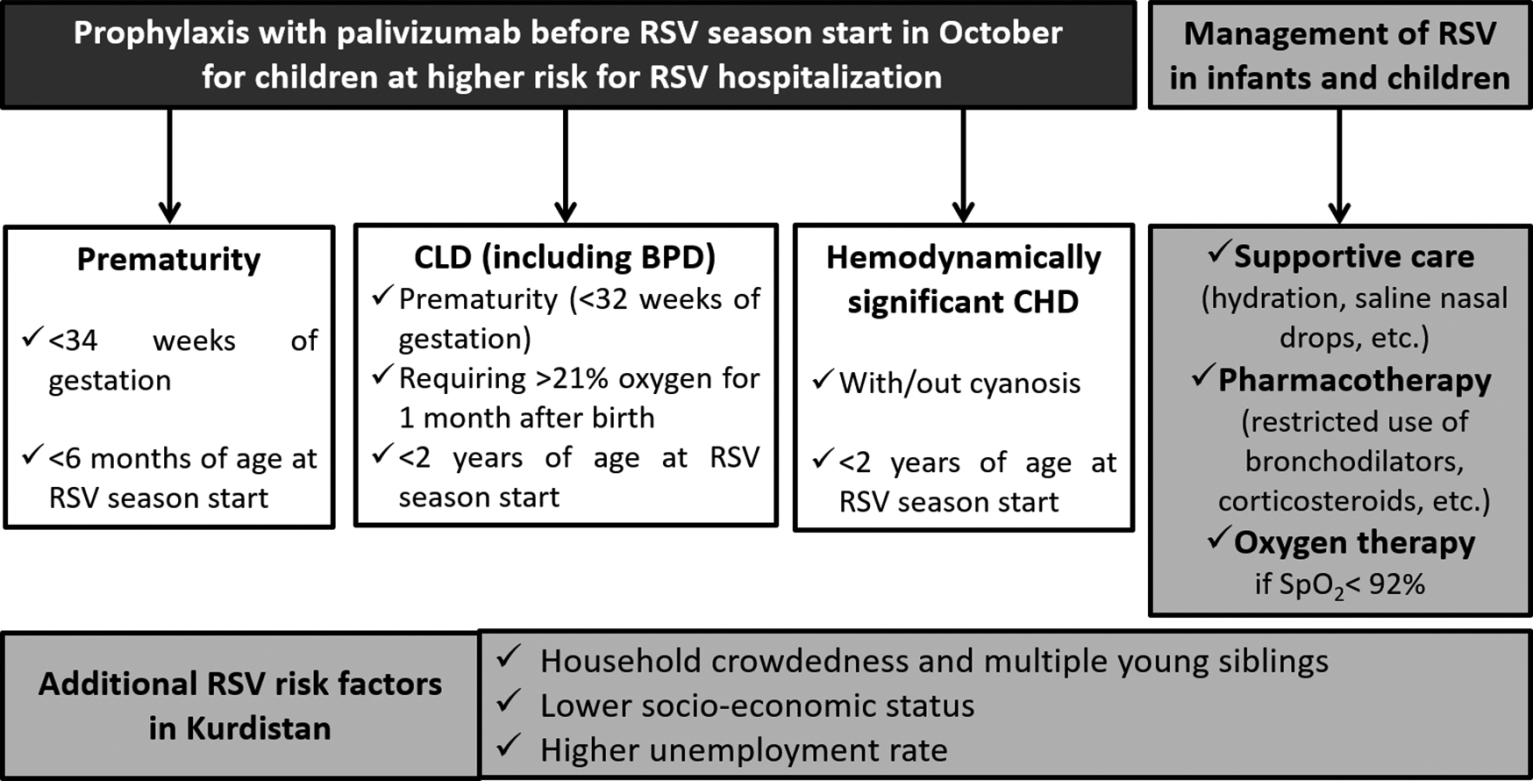
Summary recommendations for RSV management in Kurdistan. Particular risk factors to Kurdistan warrant wider eligibility criteria for immunoprophylaxis with palivizumab. While supportive care is the mainstay of treatment, pharmacotherapy should be cautiously used and oxygen therapy should be offered to all patients who struggle to maintain SpO_2_> 92%. BPD, bronchopulmonary dysplasia; CHD, congestive heart disease; CLD, congenital lung disease; RSV, respiratory syncytial virus; SpO_2_, oxygen saturation.

Based on the particular situation in Kurdistan delineated above, and the characteristics inherent to RSV infection, experts recommend the use of immunoprophylaxis to prevent infection and complications among all infants and children at risk for severe RSV disease (notably premature infants and children with CLD or CHD) and to limit RSV transmission in the community. They recommend five doses on monthly basis, at the intramuscular to anterolateral aspect of the thigh of high-risk children before the start of the RSV season. The successful development and the deployment of a RSV vaccine globally and in Kurdistan are urgently needed and eagerly awaited by pediatricians and public health experts worldwide, but until that advent, experts in Kurdistan endorse broad immunoprophylaxis of the Kurdistan population at risk for severe RSV, as summarized in Figure 1 below. Expert pediatricians also encourage staying up to date on new medications (prophylactic or therapeutic) that may be introduced to global markets, learn from the experience of other countries, and strive to evaluate novel products on the Kurdistan population.

## 6 Conclusions

Given internationally applicable guidelines and the particular situation in regions similar to Kurdistan, RSV prevention is a major medical and public health concern; especially when it exposes infants and vulnerable children to higher risk of RSV-related complications and need for hospital admission. Palivizumab immunoprophylaxis is currently the only available preventive strategy in Kurdistan and is warranted to mitigate RSV burden in the region.
